# Supplementary material for: The causal relationship between the human gut microbiota and pyogenic arthritis: a Mendelian randomization study
Source: Front Cell Infect Microbiol. 2024 Nov 26;14:1452480. doi: 10.3389/fcimb.2024.1452480 (PMC11629706; doi:10.3389/fcimb.2024.1452480)
Supplement: Supplementary file 1 [file DataSheet1.zip › mendelian test/ebi-a-GCST90027665.csv_leave_one_out.pdf]

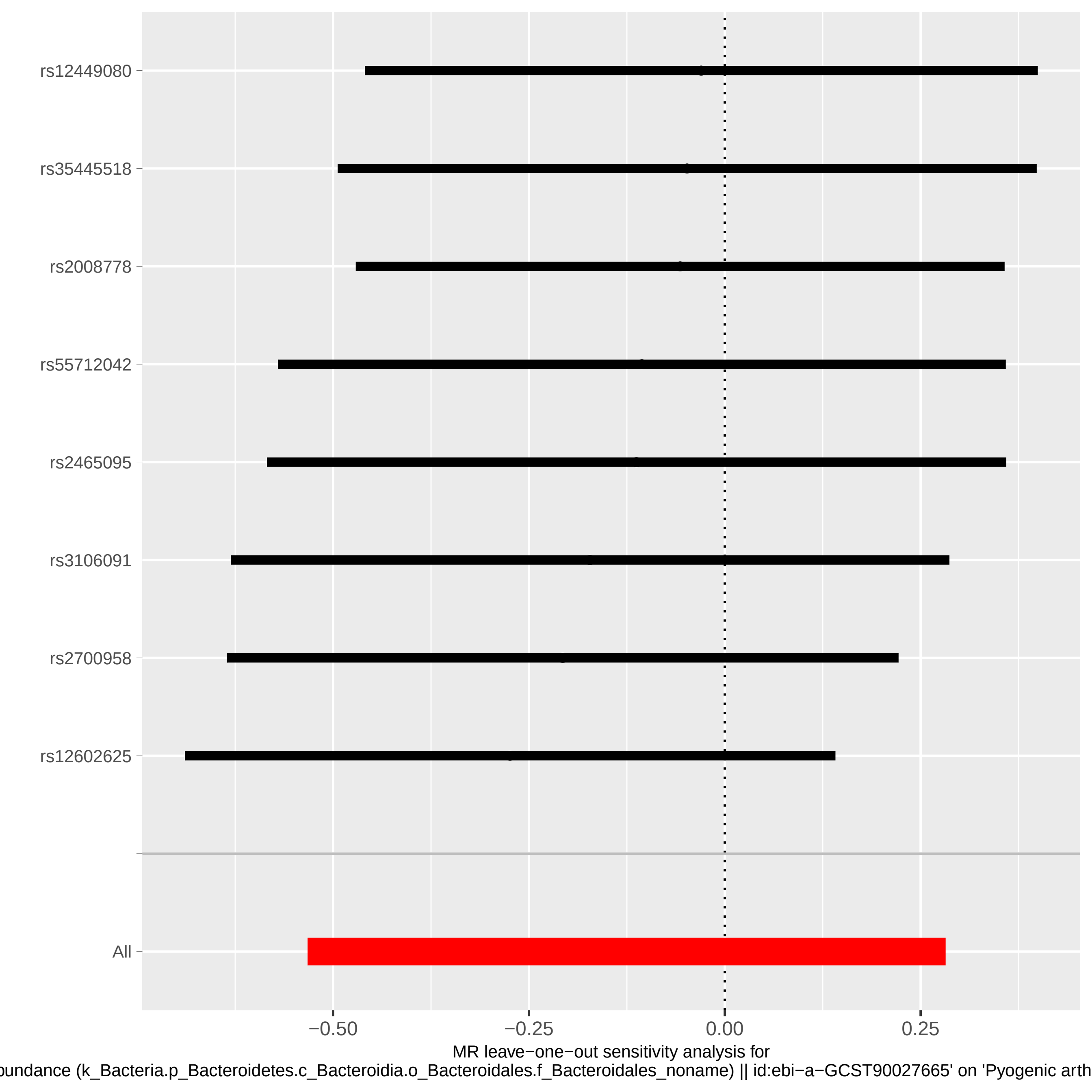
rs12449080
rs35445518
rs2008778
rs55712042
rs2465095
rs3106091
rs2700958
rs12602625
All
−0.50
−0.25
0.00
0.25
MR leave−one−out sensitivity analysis for
oundance (k_Bacteria.p_Bacteroidetes.c_Bacteroidia.o_Bacteroidales.f_Bacteroidales_noname) || id:ebi−a−GCST90027665' on 'Pyogenic arth
